# An integrated transcriptome and microbial community analysis reveals potential mechanisms for increased immune responses when replacing silybum marianum meal with soybean meal in growing lambs

**DOI:** 10.3389/fmicb.2023.1093129

**Published:** 2023-03-02

**Authors:** Tianxi Zhang, Yanbo Ren, Chao Yang, Kefyalew Gebeyew, Min Gao, Zhixiong He, Zhiliang Tan

**Affiliations:** ^1^CAS Key Laboratory for Agro-Ecological Processes in Subtropical Region, National Engineering Laboratory for Pollution Control and Waste Utilization in Livestock and Poultry Production, Hunan Provincial Key Laboratory of Animal Nutritional Physiology and Metabolic Process, Institute of Subtropical Agriculture, The Chinese Academy of Sciences, Changsha, China; ^2^University of Chinese Academy of Science, Beijing, China; ^3^School of Animal Science, Inner Mongolia Agricultural University, Hohhot, China; ^4^Inner Mongolia Academy of Agricultural and Animal Husbandry Sciences, Hohhot, China

**Keywords:** silybum marianum meal, soybean meal, lambs, cytokines, microbial community, transcriptome

## Abstract

Silybum marianum meal is a by-product that remains silymarin complex and is perceived as a potential-protein source. The potential and its mechanism of silybum marianum meal as a protein supplement in ruminants were evaluated by testing the growth performance, biochemical parameters, cytokine levels, gut transcriptome and microbial community profiles. Forty-two male Hulunbeier growing lambs (aged about 3-month-old; averaged body weight of 21.55 kg) were randomly divided into the CON (with 10% soybean meal) and SIL groups (with 10% silybum marianum meal). There was no significant difference in growth performance, feed intakes, or serum biochemical parameters between CON and SIL. The serum levels of IL-1β, TNF-α, TGF-β, HGF, and VEGF were all increased (*p* < 0.05) in the SIL group as compared with the CON group. Transcriptome gene set enrichment analysis (GSEA) revealed that the core genes in the rumen from SIL group were enriched with fructose and mannose metabolism, while the core genes in the ileum were enriched for three biological process, including digestive tract development, positive regulation of MAPK cascade, and regulation of I-kappaB kinase/NF-kappaB signaling. The 16S rDNA results showed that the relative abundance of *Bacteroidetes*, *Firmicutes*, *Synergistetes*, and *Verrucomicrobia* in the rumen from SIL group was significantly higher than that in CON group (*p* < 0.05), whereas *Proteobacteria* was significantly lower than that in CON group (*p* < 0.05). The LEfSe analysis showed that the genera *Pyramidobacter*, *Saccharofermentans*, *Anaerovibrio*, *Oscillibacter* and *Barnesiella* were enriched in the rumen from SIL group, whereas *Sharpea* was enriched in the CON group (LDA > 2). In the ileum, there were no significant differences in the phylum-level classification of microbes observed. At the genus level, the relative abundances of *Bifidobacterium* and *Ruminococcus* in the ileum from SIL group were significantly higher than that in the CON group (*p* < 0.05), whereas the relative abundance of *Clostridium_XI* was lower (*p* < 0.05). Correlation analysis showed that *Clostridium_XI* was negatively correlated with VEGF, TGF-β, TNF-α and HGF (*p* < 0.05). Core genes *BMP4* and *CD4* were negatively correlated with *Clostridium_XI* (*p* < 0.05). Our results indicated that supplementing silybum marianum meal as a replacement for soybean meal resulted in increased cytokines production without affecting growth performance in growing lambs, and the enrichment of immune-related genes and altered microbial community in the ileum were contributed to the increased immune responses.

## 1. Introduction

The livestock industry is struggling to access quality protein feed that could satisfy animal body requirements at an affordable price, in particular in developing countries. With the current trends of rising prices of soybean meal, it is essential to develop locally available low-cost protein supplements. Silybum marianum is a kind of medicinal herb with potential health effects, which is widely grown in Asia and Latin America. The silymarin, a main bioactive component of silybum marianum, contains an isomeric mixture of unique flavonoid complexes and has hepatic protection, detoxification and anti-oxidation functions (Tajmohammadi et al., 2018). The application of silymarin in livestock has received considerable attention due to the advancement of extraction technology and its role in promoting growth. Silymarin has been shown to have a positive effect on the production and carcass plasma profiles in laying hens (Šťastník et al., 2019), horse (Dockalova et al., 2021), and pigs (Grela et al., 2020). In addition, silybum marianum meal is a by-product of silymarin production (Dehghan et al., 2011). Due to its high protein content, it has a potential to use as an unconventional feed resource (Fan et al., 2019). However, mature silybum marianum contains nitrite, which is supposed to have a toxic effect in animal (Arviv et al., 2016), resulting in increased inflammatory responses. In addition, silybum marianum meal contains a high fiber level (Stastnik et al., 2020), which may be beneficial for ruminants theoretically. The potential and its mechanism of silybum marianum meal as a protein supplement in ruminants remain uncovered.

With the development of high-throughput sequencing technology, multi-omics approach was widely used to explore the interaction between microbiota and host in response to variable phenotypes at a molecular level. The objectives of this study were: (1) to evaluate the effects of substituting soybean meal with silybum marianum meal on the growth performance, biochemical parameters and cytokine levels, (2) to explore the potential mechanism in response to altered phenotypes by using an integrated transcriptome and microbial community analysis.

## 2. Materials and methods

### 2.1. Animals, diets, and experimental design

The experimental procedures of this study were carried out in accordance with the guidelines of the Animal Ethics Committee of the Institute of Subtropical Agriculture, Chinese Academy of Sciences. A total of 42 healthy 3-month-old Hulunbeier lambs (averaged with 21.55 kg) with similar body weight were selected and randomly divided into two groups (initial weight was tested for non-significant differences between groups), with 21 sheep in each group. The experimental animals were divided into the CON (supplemented with 10% soybean meal) and SIL groups (supplemented with 10% silybum marianum meal). The compositions and nutrient levels of the experimental diets are shown in Table 1. The experiment lasted for 90 days, including the pre-feeding period of 15 days and the trial period of 75 days. During the pre-feeding period, the experimental animals were ear tagged, dewormed, weighed, and vaccinated according to the normal immunization program for lamb fattening. The sheep were fed and watered three times a day, at 6:00, 11:00 and 18:00, respectively, to ensure that there was a surplus of diet and water. The same diet was fed during the pre-feeding period and the trial period.

**Table 1:** Ingredient and chemical composition (% DM) of the experimental diets.

Item	Treatment	
	CON	SIL
Ingredients		
Corn	30.51	30.51
Barley	5.57	5.57
Bran	8.72	8.72
Soybean meal	10.00	0
silybum marianum meal	0	10.00
Trace elements with vitamin premix^1^	2.00	2.00
Sodium bicarbonate	0.70	0.70
Salt	0.50	0.50
Calcium carbonate	1.00	1.00
Sugar beet molasses	1.00	1.00
Alfalfa hay	30.00	30.00
Wheat stalk	10.00	10.00
Total	100.00	100.00
Chemical composition,(% of DM)		
DM	94.82	94.06
CP	14.82	12.83
ADF	6.66	6.81
NDF	22.66	23.93
Ca	0.33	0.53
P	0.31	0.35
ME(MJ/kg)	10.46	10.11

CON = supplemented with 10% soybean meal; SIL = supplemented with 10% silybum marianum meal; DM = dry matter; ME = metabolizable Energy; CP = crude protein; ADF = acid detergent fiber; NDF = neutral detergent fiber; Ca = calcium; P = phosphorus.

1 The premix provides per kilogram of ration: Fe 221.31 mg, Cu 16.23 mg, Zn 71.31 mg, Mn 71.31 mg, I 1.23 mg, Se 1.70 mg, Co 0.54 mg, VA 16250 IU, VD 5000 IU, VE 40 IU.

### 2.2. Sample collection

All the experimental sheep were weighed every 2 weeks before morning feeding from the beginning of the experimental period, the data were recorded, and the daily weight gain was calculated. The amount of feed was recorded daily, and the leftover was recorded before feeding the following day. The remaining feed was cleared afterward, and a new feed was added. Average daily feed intake = total feed intake/number of experimental days; Feed weight ratio (F/g) = average daily intake / average daily gain.

At the end of the animal test, seven tested sheep in each group were randomly selected for blood collection by jugular venipuncture before morning feeding. The blood was collected with a 10 ml BD Vacutainer serum tube and stored at 4°C for 30 min, then centrifuged at 3,000 rpm/min for 15 min to separate the serum. The collected supernatant was placed in a 1.5 ml centrifuge tube and stored at −20°C.

After dissection, the rumen was opened, and the rumen digesta collected. Then, rumen abdominal sac (5 × 5 cm) was collected. The ileum digesta and ileum tissue were collected from 50 cm proximal to the junction of the ileum and the cecum. Tissue samples were washed with pre-cooled phosphate buffer during collection. After collection, digesta and tissue samples were snap-frozen in liquid nitrogen and stored in an ultra-low temperature freezer at −80°C. Tissue samples were used for RNA extraction and sequencing, and digesta samples were used for 16S rDNA gene sequencing and bioinformatics analysis. Since the rumen and ileum were vital organs for feed digestion and immune functions (Mowat and Agace, 2014; Na and Guan, 2022), their tissue and digest were selected for further transcriptome and microbial community analysis.

### 2.3. Analysis of biochemical parameters and cytokine levels in blood

Serum biochemical parameters, including total protein (TP), high-density lipoprotein (HDL), glutamic aminotransferase (ALT), glutamic oxalacetic aminotransferase (AST), lactate dehydrogenase (LDH), glucose (GLU), triglycerides (TG), total cholesterol (CHOL), amylase (AMS), blood ammonia (NH3L), total bilirubin (TBIL), and c-reactive protein (CRP), were measured using an automated biochemical instrument (Cobasc311, Roche, Basel, Switzerland). Serum cytokine indicators, including interleukin 1β (IL-1β), interleukin 2 (IL-2), tumor necrosis factor (TNF-α), transforming growth factor beta (TGF-β), hepatocyte growth factor (HGF), vascular endothelial growth factor (VEGF), insulin-like growth factor 1 (IGF-1) were measured using commercial sheep-specific ELISA kits(Jiangsu Meimian industrial Co., Ltd., Yancheng, China).

### 2.4. Rumen and ileum RNA sequencing and bioinformatic analysis

Total RNA was extracted from 14 rumen and 14 ileum tissues using Trizol (Invitrogen, Carlsbad, CA, United States) according to the manual. Subsequently, total RNA was characterized and quantified using a NanoDrop and Agilent 2,100 Bioanalyzer (Thermo Fisher Scientific, MA, United States).

Oligo (dT)-linked magnetic beads were used to purify mRNA. The purified mRNA was split into small pieces at the appropriate temperature using a fragmentation buffer. First-strand cDNA was then generated using random hexamer-triggered reverse transcription, followed by the synthesis of second-strand cDNA. After which, A-Tailing Mix and RNA Index Adapters were added by incubation to conclude the repair. The cDNA fragment obtained in the previous step was amplified by PCR and the product was purified by Ampure XP Beads and then dissolved in EB solution. The product was validated for quality control on an Agilent Technologies 2,100 Bioanalyzer. The double-stranded PCR products from the previous step were heat denatured and cyclized by splinting oligonucleotide sequences to obtain the final library. Single-stranded circular DNA (ssCirDNA) was formatted as the final library. The final library was amplified with phi29 to make DNA nanoballs (DNBs) with more than 300 copies of one molecule, DNBs were loaded into patterned nanoarrays, and single-end 50-base reads were generated on the BGIseq500 platform (BGI-Shenzhen, China).

The sequencing data was filtered with SOAPnuke (v1.5.2) (Li et al., 2008): (1) to remove reads containing sequencing adapters; (2) to remove reads with a low-quality base ratio (base quality less than or equal to 5) greater than 20%; (3) Remove reads with a ratio of unknown bases (‘N’ bases) greater than 5%, after which clean reads are obtained and saved in FASTQ format. These clean reads were mapped onto the sheep reference genome (GCF_002742125.1_Oar_rambouillet_v1.0) using HISAT2 (v2.0.4) (Kim et al., 2015). Bowtie2 (v2.2.5) (Langmead and Salzberg, 2012) was applied to align the clean reads to the reference coding genome, and then RSEM (v1.2.12) (Li and Dewey, 2011) was used to calculate gene expression levels. Transcripts Per Kilobase of exon model per Million mapped reads (TPM) were used to estimate the amount of gene expression in each sample. The Gene Set Enrichment Analysis (GSEA) algorithm was used to identify the pathways, biological processes, or functional components of genes with the most significant changes in expression in the CON and SIL samples (|NES| > 1, Nominal value of p:<0.05, FDR q-value:<0.25, https://www.gsea-msigdb.org/gsea/index.jsp).

### 2.5. Sequencing and bioinformatics analysis of 16SrDNA gene of rumen and ileum microorganisms

Microbial DNA was extracted from 14 rumen digest samples and 14 ileum digest samples using MagPure Stool DNA KF kit B (Magen, China) according to the manufacturer’s instructions. DNA was quantified with a Qubit Fluorometer by using Qubit dsDNA BR Assay kit (Invitrogen, United States) and the quality was checked by running aliquot on 1% agarose gel. The variable region V3-V4 of the bacterial 16S rRNA gene was amplified using the condensed PCR primers 341F (5’-ACTCCTACGGGAGGCAGCAG-3′) and 806R (5’-GGACTACHVGGGTWTCTAAT-3′). Forward and reverse primers were labeled with Illumina adapters, pads and splice sequences. PCR enrichment was performed in a 50 μ L reaction containing a 30 ng template, fusion PCR primers and PCR master mix. PCR cycling conditions were as follows: 94°C for 3 min, 94°C for 30 s, 56°C for 45 s, 72°C for 45 s, 30 cycles, 72°C for 10 min final extension for 10 min. PCR products were purified with AmpureXP beads and eluted in elution buffer. Libraries were characterized by Agilent 2,100 Bioanalyzer (Agilent, United States). Validated libraries were used for sequencing on the Illumina MiSeq platform (BGI, Shenzhen, China) following Illumina’s standard procedure and generating 2 × 300 bp double-end reads.

The raw reads were filtered to remove splices and low quality and ambiguous bases, and then double-ended reads were added to the tags by the Fast Length Adjustment of Short reads program (FLASH, v1.2.11) (Magoc and Salzberg, 2011) to obtain the tags. The tags were clustered into OTUs with a cutoff of 97% using UPARSE software (v7.0.1090) (Edgar, 2013) and the chimeric sequences were compared to the Gold database for detection using UCHIME (v4.2.40) (Edgar et al., 2011). Then, OTU representative sequences were classified using the Ribosome Database Project (RDP) classifier v.2.2 with a minimum confidence threshold of 0.6 and trained on the Greengenes database v201305 using QIIME v1.8.0 (Caporaso et al., 2010). All Tags were compared with OTUs using USEARCH_global (Edgar, 2010) to obtain a statistical table of OTU abundance for each sample. The data were analyzed for diversity indices based on OTUs, including Chao1, ACE, Simpson and Shannon indices. Beta diversity analysis was performed using principal coordinate analysis (PCoA) with weighted UniFrac distances. Linear discriminant analysis (LDA) effect size (LEfSe) tools were applied to identify different taxa between CON and SIL groups by using an online software package (LC Bio-Technology Co., Ltd., Hangzhou, China) with thresholds (*p* < 0.05 and LDA > 2).

### 2.6. Statistical analysis

Firstly, Shapiro–Wilk and Levene’s tests were employed to confirm the normality and homoscedasticity of data, respectively. The serum biochemical and immune indices, and alpha-diversity index results were analyzed using the independent-sample t-test in SPSS software (SPSS version 25.0, SPSS, Inc.). The relative abundance bacteria at the phylum and genus levels was assessed using Wilcoxon rank-sum test. Data are presented as means ± SEM. *p* < 0.05 was regarded as statistically significant, and 0.05 ≤ *p* < 0.10 was regarded as a statistical tendency. The correlation analysis was performed using Spearman’s rank correlation coefficient in R package and the network was constructed on the Omics-studio (LC-Bio Technology Co., Ltd., Hangzhou, China).

## 3. Results

### 3.1. Effects of replacing soybean meal with silybum marianum meal on growth performance, biochemical parameters, and cytokine levels in growing lambs

No difference (*p* > 0.05) was observed between SIL and CON for final body weight, feed average daily gain, or feed intake (Table 2). Most of the biochemical parameters were not affected by replacing soybean meal with silybum marianum meal except that the TP tended to decrease (*p* = 0.08) and the AST tended to increase (*p* = 0.06; Table 3).

**Table 2:** Effect of silybum marianum meal as a replacement for soybean meal on feed intake and growth performance of lambs.

Item	Treatment	SEM	*p*-value	CON (*n* = 21)	SIL (*n* = 21)
Initial weight (Kg)	21.09	22.00	0.26	0.307
Final weight (Kg)	31.61	32.92	0.39	0.398
Average dry matter intake (Kg)	1.29	1.22	0.02	0.477
Average daily gain (Kg)	0.167	0.173	0.01	0.771
Ratio of feed to gain (F/G)	8.20	7.48	0.18	0.272

CON = supplemented with 10% soybean meal; SIL = supplemented with 10% silybum marianum meal.

Values are expressed as means ± SEM, *n* = 21. *p* < 0.05 was regarded as statistically significant, and 0.05 < *p* < 0.10 was regarded as a statistical tendency.

Average daily feed intake = total feed intake / test days; Ratio of feed to gain (F/G) = average daily feed intake/average daily gain.

**Table 3:** Effect of silybum marianum meal as a replacement for soybean meal on serum biochemical parameters in lambs.

Item	Treatment		SEM	*p*-value
	CON (*n* = 7)	SIL (*n* = 7)		
TP (g/L)	81.59	76.80	0.89	0.083
HDL (mmol/L)	1.28	1.37	0.04	0.729
ALT (U/L)	22.79	23.93	1.25	0.936
AST (U/L)	167.78	177.44	8.15	0.060
LDH (U/L)	816.33	772.25	20.34	0.658
GLU (mmol/L)	5.40	5.27	0.28	0.751
TG (mmol/L)	0.46	0.41	0.02	0.656
CHOL (mmol/L)	1.88	1.98	0.05	0.473
AMS (U/L)	12.11	11.00	0.72	0.681
NH3L (mol/L)	552.89	534.89	16.21	0.854
TBIL (mmol/L)	3.41	3.42	0.17	0.315
CRPL3 (mg/L)	3.89	3.86	0.01	0.729

CON = supplemented with 10% soybean meal; SIL = supplemented with 10% silybum marianum meal; TP = total protein; HDL = high-density lipoprotein; ALT = alanine transaminase; AST = aspartate transaminase; LDH = lactate dehydrogenase; GLU = glucose; TG = triglycerides; CHOL = total cholesterol; AMS = amylase; NH_3_L = blood ammonia; TBIL = total bilirubin; CRP=C reactive protein.

Values are expressed as means ± SEM, *n* = 7.

Serum cytokines were determined to assess the effects of the immune response when replacing silybum marianum meal (Table 4). The levels of IL-1β and TNF-α were significantly increased in SIL as compared with CON (*p* < 0.05). Similarly, lambs in SIL had higher concentrations (*p* < 0.05) of TGF-β, HGF and VEGF than those in CON. The concentrations of IL-2 (*p* = 0.083) and IGF-1 (*p* = 0.075) tended to be higher in SIL as compared with CON.

**Table 4:** Effect of silybum marianum meal as a replacement for soybean meal on serum cytokine levels in lambs.

Item	Treatment		SEM	*p*-value
	CON (*n* = 7)	SIL (*n* = 7)		
IL-1β (pg/mL)	418.06^b^	546.65^a^	20.01	0.018
IL-2 (pg/mL)	683.43	788.85	19.51	0.083
TNF-α (pg/mL)	108.12^b^	144.99^a^	5.48	0.002
TGF-β (ng/mL)	31.21^b^	39.41^a^	0.91	<0.001
HGF (pg/mL)	1242.97^b^	1534.02^a^	44.11	0.017
VEGF (pg/mL)	109.26^b^	132.16^a^	3.08	<0.001
IGF-1 (ng/mL)	1236.98	1381.17	32.10	0.075

CON = supplemented with 10% soybean meal; SIL = supplemented with 10% silybum marianum meal; IL-1β = interleukin 1β; IL-2 = interleukin 2; TNF- α = tumor necrosis factor; TGF- β = transforming growth factor β; HGF=Hepatocyte growth factor; VEGF = vascular endothelial cell growth factor; IGF-1 = insulin-like growth factor 1.

Values are expressed as means ± SEM, *n* = 7. *p* < 0.05 was regarded as statistically significant, and 0.05 < *p* < 0.10 was regarded as a statistical tendency.

Values in the same row with different superscript letters (a, b) differ significantly (*p* < 0.05).

### 3.2. Effects of replacing soybean meal with silybum marianum meal on transcriptome profiles in rumen and ileum of growing lambs

The transcriptomes of the rumen and ileum tissues were evaluated to explore the potential mechanism in response to the altered cytokines in the blood. RNA-seq of the rumen tissue in the CON and SIL groups yielded approximately 23.95 and 23.38 million clean reads, of which 95.67 and 95.49% were mapped to the sheep reference genome (Supplementary file 2; Sheet 1). About 17,682 and 17,958 expressed genes were detected in the rumen of the CON and SIL groups, respectively (Supplementary file 2; Sheet 2). RNA-seq of the ileum tissue from the CON and SIL groups yielded approximately 23.55 and 23.75 million clean reads, 94.55 and 94.37% of which were mapped to the sheep reference genome (Supplementary file 2; Sheet 3). 16,127 and 16,092 expressed genes were detected in ileum tissue from the CON and SIL groups, respectively (Supplementary file 2; Sheet 4).

**Figure 1:**
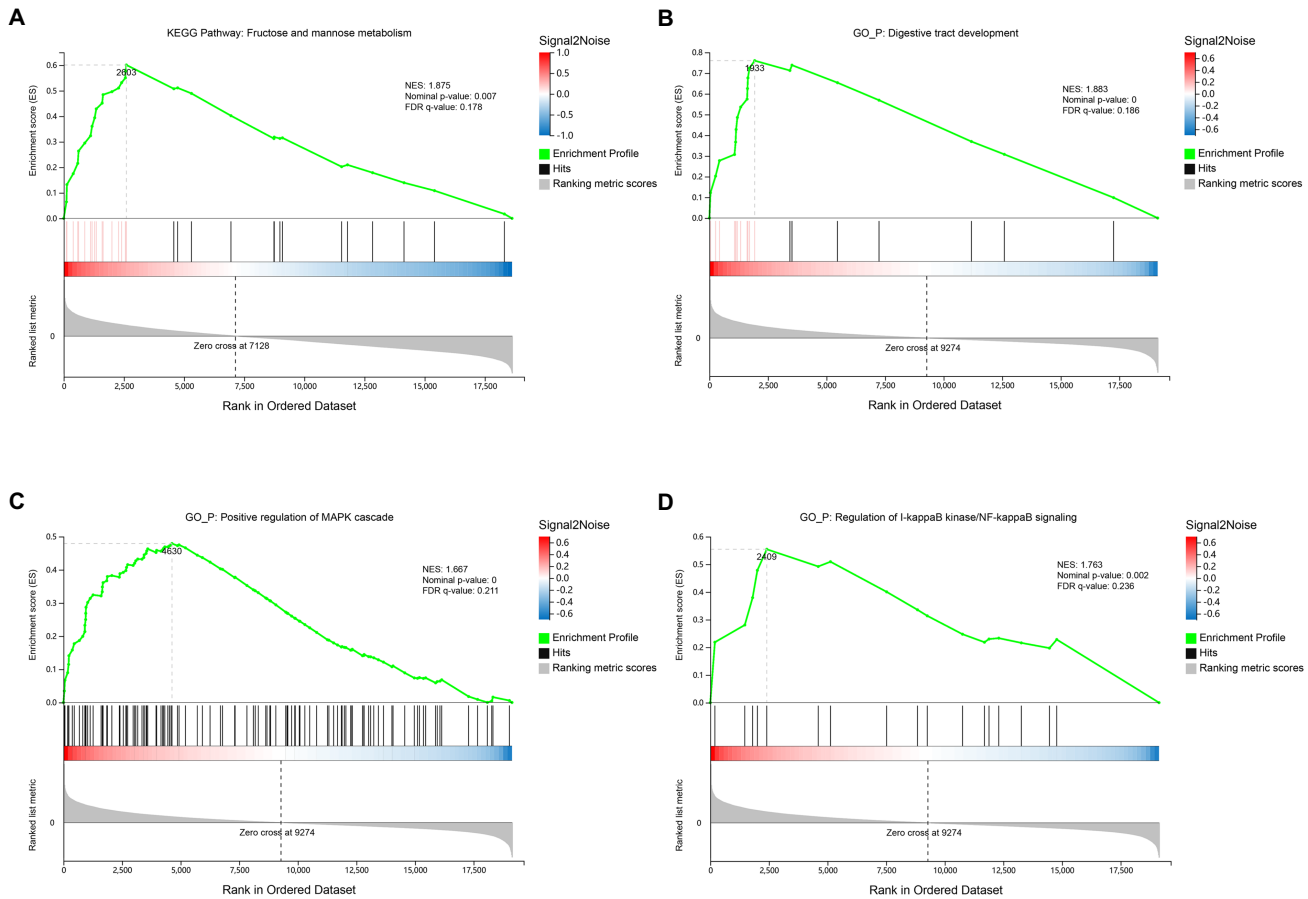
Transcriptome gene set enrichment analysis (GSEA) of rumen and ileum tissue of lambs in the CON and SIL groups. **(A)**: Gene set enriched in Fructose and mannose metabolism pathway of rumen; **(B)**: Gene set enriched in digestive tract development biological process of ileum; **(C)**: Gene set enriched in positive regulation of MAPK cascade biological process of ileum; **(D)**: Gene set enriched in regulation of I-kappaB kinase/NF-kappaB signaling biological process of ileum. Screening criteria for significant gene sets included adj. *p*-value <0.05 and FDR < 0.25. NES: normalized enrichment score.

Since the number of differential expressed genes observed in the rumen or ileum between groups was small (Supplementary file 4), GSEA algorithm was used to further identify the pathways, biological processes or functional components of genes with the most significant changes in expression. Results showed that the rumen of the SIL was enriched with fructose and mannose metabolism-related genes in the Kyoto Encyclopedia of Genes and Genomes (KEGG) (NES: 1.875, Nominal value of p: 0.007, FDR q-value: 0.178) (Figure 1A; Supplementary file 3; Sheet 1). Ileum of the SIL was enriched for three biological process-related genes in the Gene Ontology (GO), including digestive tract development (NES: 1.883, Nominal value of *p*: <0.001, FDR *q*-value: 0.186) (Figure 1B; Supplementary file 3; Sheet 2), positive regulation of MAPK cascade (NES: 1.667, Nominal value of p: <0.001, FDR q-value: 0.211) (Figure 1C; Supplementary file 3; Sheet 3) and regulation of I-kappaB kinase/NF-kappaB signaling (NES: 1.763, Nominal value of p: 0.002, FDR q-value: 0.236) (Figure 1D; Supplementary file 3; Sheet 4).

### 3.3. Effects of replacing soybean meal with silybum marianum meal on microbial community in rumen and ileum of growing lambs

**Figure 2:**
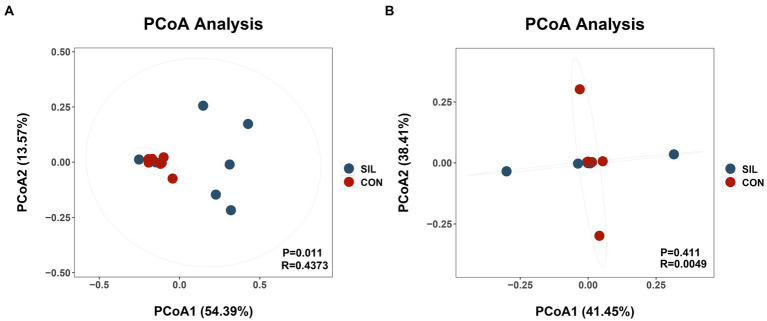
Beta diversity of the microbial communities in the rumen digesta of lambs in the CON and SIL groups. Principal coordinate analysis (PCoA) of weighted UniFrac distance metric of rumen **(A)** and ileum **(B)**. CON = supplemented with 10% soybean meal; SIL = supplemented with 10% silybum marianum meal. *p* < 0.05 was regarded as statistically significant.

16SrDNA gene sequencing was used to determine the microbial community composition in the rumen and ileum. As shown in Table 5, an average of 511.13 and 658.86 OTUs with 97% similarity per sample were obtained in the rumen of CON and SIL groups, respectively, which was significantly higher in SIL as compared to CON (*p* < 0.05). As shown by the Coverage index of the two groups, the coverage of each sample was more than 99%, thus accurately reflecting the diversity of microbial community species and structure in the rumen. Based on the OTU clustering results for α-diversity analysis, the ACE index, Chao1 index, and Shannon index of the SIL group were significantly higher than those of the CON group (*p* < 0.05). However, the Simpson index of SIL group was significantly lower than that of the CON group (*p* < 0.05). The results of PCoA analysis with weighted distance metric showed that the rumen community composition of the CON group was clearly separated (*p* < 0.05 and R > 0.25) from the SIL group (Figure 2A).

**Table 5:** Effect of silybum marianum meal as a replacement for soybean meal on the alpha diversity index of the rumen and ileum digesta of lambs.

Item	Treatment		SEM	*p*-value
	CON (*n* = 7)	SIL (*n* = 7)		
Rumen				
OTUs	511.130^b^	658.860^a^	21.08	0.001
Chao1 index	673.390^b^	807.050^a^	24.06	0.008
ACE index	694.410^b^	818.565^a^	22.11	0.004
Shannon index	2.970^b^	3.963^a^	0.12	0.004
Simpson index	0.180^a^	0.091^b^	0.01	0.040
Good’s coverage	0.996^a^	0.995^b^	0.001	0.005
Ileum				
OTUs	416.857	550.625	66.990	0.694
Chao1 index	600.729	723.454	72.896	0.613
ACE index	700.496	778.302	65.315	0.694
Shannon index	2.234	2.781	0.263	0.779
Simpson index	0.210	0.192	0.025	0.955
Good’s coverage	0.997	0.996	0.000	0.536

CON = supplemented with 10% soybean meal; SIL = supplemented with 10% silybum marianum meal.

Values are expressed as means ± SEM, *n* = 7. *p* < 0.05 was regarded as statistically significant, and 0.05 < *p* < 0.10 was regarded as a statistical tendency.

a Means with different superscripts differ (*p* < 0.05).

b Means with different superscripts differ (*p* < 0.05).

**Figure 3:**
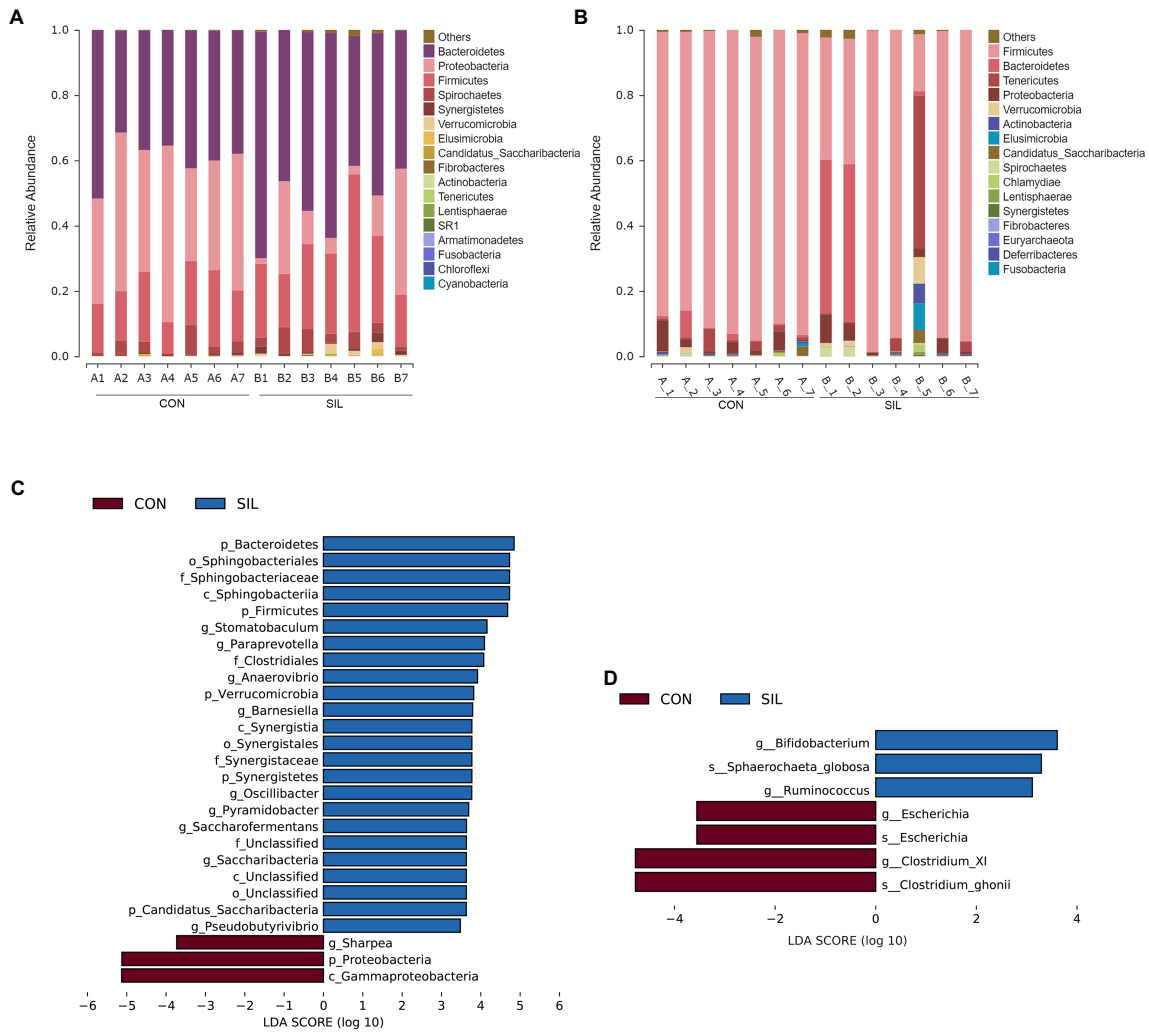
Taxonomic analysis of the microbial communities in the rumen and ileum digesta samples of lambs in the CON and SIL groups. The relative abundance of microbial composition at the phylum level in the rumen **(A)** and ileum **(B)**; Linear discriminant analysis (LDA) effect size linear discriminant analysis (LEfSe) of the rumen **(C)** and ileum **(D)** CON = supplemented with 10% soybean meal; SIL = supplemented with 10% silybum marianum meal.

Taxonomic analysis revealed 14 phyla co-identified by the two groups in the rumen (Figure 3A). The predominant phyla in the rumen digesta were *Bacteroidetes*, *Proteobacteria*, and *Firmicutes*. The relative abundances of those top three phyla were 39.71, 39.77 and 16.33% in the CON group and 52.27, 14.10, and 25.61% in the SIL group, respectively. The relative abundances of *Bacteroidetes* and *Firmicutes* in the SIL group were significantly higher than those in the CON group (*p* < 0.05), but *Proteobacteria* were significantly lower than those in the CON group (*p* < 0.05) (Supplementary file 1; Table 1). In addition, the relative abundances of *Synergistetes* and *Verrucomicrobia* in the SIL group were also significantly higher than those in the CON group (*p* < 0.05) (Supplementary file 1; Table 1). At the genus level, the relative abundances of *Pyramidobacter* and *Saccharofermentans* were higher in the SIL group as compared with the CON group (*p* < 0.05). Conversely, the relative abundance of *Sharpea* was lower (*p* < 0.05) in the SIL group than in the CON group (Supplementary file 1; Table 2). The results of LEfSe analysis showed that the genera *Pyramidobacter*, *Saccharofermentans*, *Anaerovibrio*, *Oscillibacter* and *Barnesiella* were enriched in the SIL group, whereas *Sharpea* was enriched in the CON group (LDA > 2) (Figure 3B).

In the ileum, an average of 416.85 and 550.62 OTUs with 97% similarity per sample were obtained in the CON and SIL groups, respectively. The coverage of each sample was more than 99%, as shown by the Coverage index of both groups, thus accurately reflecting the diversity of microbial community species and structures in the ileum. Based on the OTU clustering results for α-diversity analysis, no significant differences in alpha and β diversity indexes were observed between the two groups (Figure 2B).

Taxonomic analysis revealed that ileum microbes were co-annotated into 15 phyla between the two groups (Figure 3C). *Firmicutes* was the most dominant phylum followed by *Bacteroidetes*. There were no significant differences at the phylum level between the two groups (Supplementary file 1; Table 3). At the genus level, at least one group of genera with a relative abundance greater than 0.1% in both groups was annotated to 43 (Supplementary file 1; Table 4). The relative abundances of *Bifidobacterium* and *Ruminococcus* in the SIL group were significantly higher than in the CON group (*p* < 0.05) (Supplementary file 1; Table 4). Conversely, the relative abundance of *Clostridium_XI* was lower (*p* < 0.05) in the SIL group than in the CON group (Supplementary file 1; Table 4). In addition, the LEfSe analysis results showed that the genera *Bifidobacterium* and *Ruminococcus* were dominant in the SIL group, and *Clostridium_XI* also was enriched in the CON group (LDA > 2) (Figure 3D).

### 3.4. Correlation between serum cytokines, transcriptional core genes and relative abundance of Top 20 genera in the ileum

**Figure 4:**
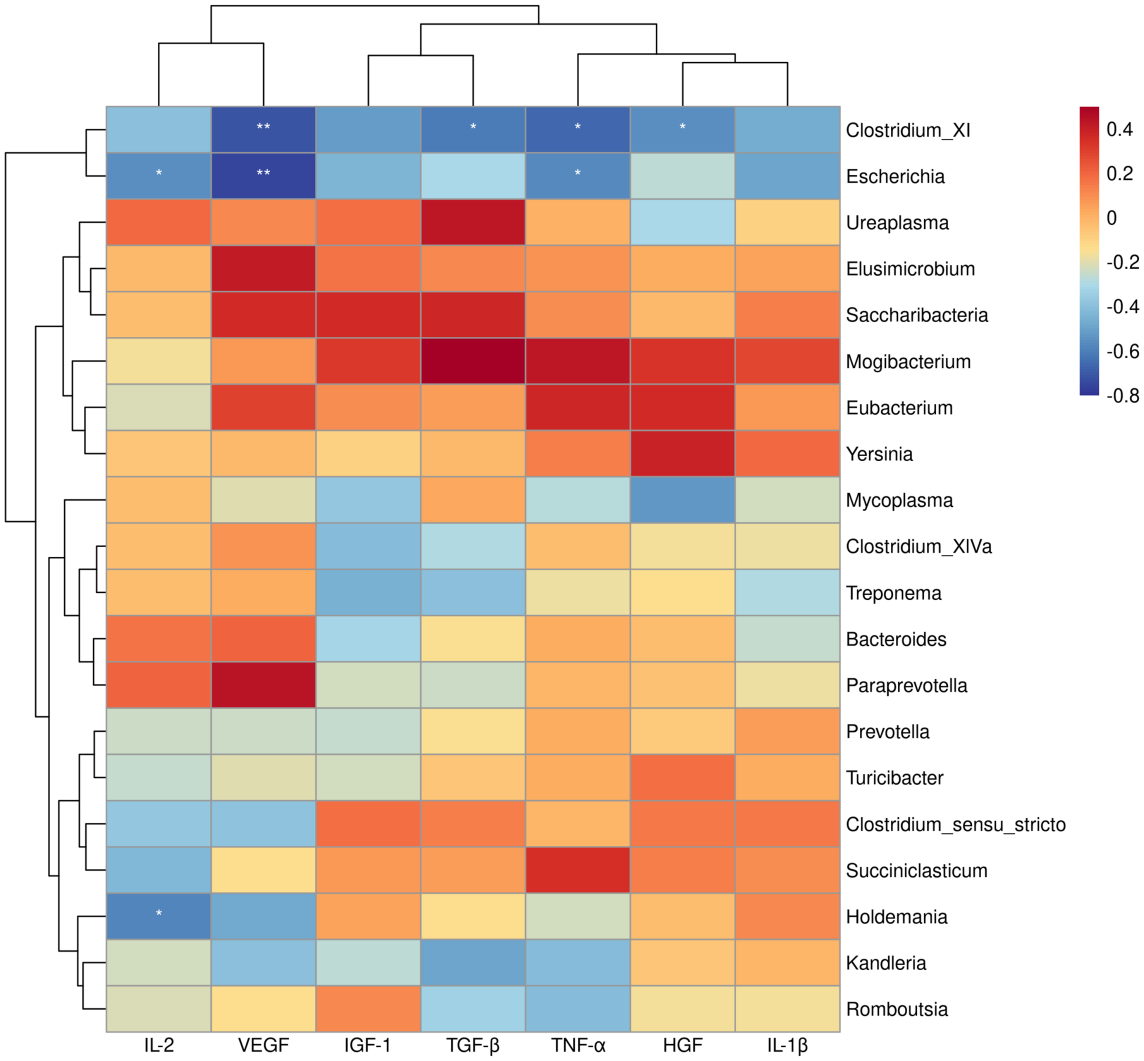
Heatmap of spearman’s correlation between serum cytokines and the relative abundance of top 20 bacteria at genus level in the ileum. The blue suggests a negative correlation, and the red suggests a positive correlation. *p < 0.05, **p < 0.01.

**Figure 5:**
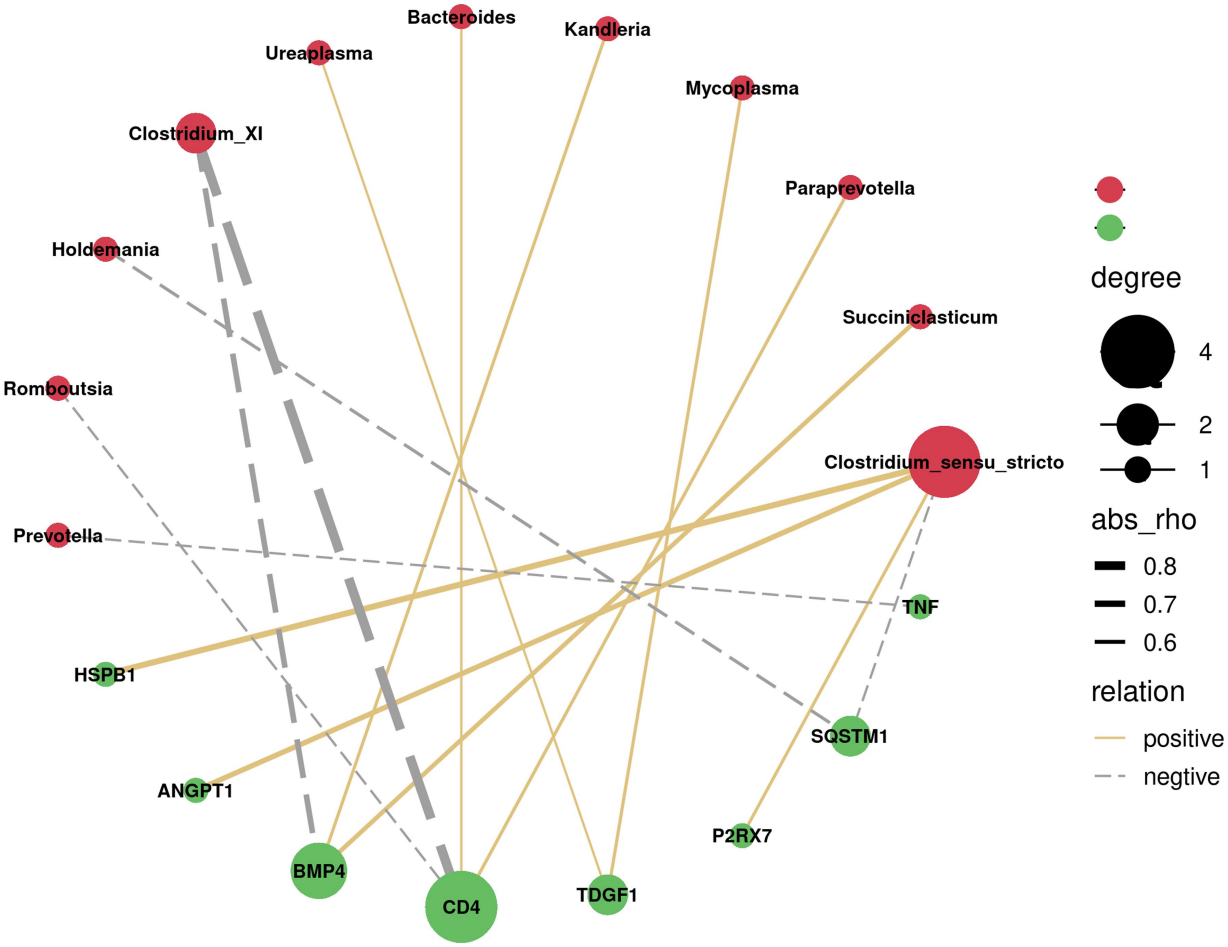
Correlation network analysis between the core genes (green circles) of positive regulation of MAPK cascade and regulation of I-kappaB kinase/NF-kappaB signalling and the predominant bacteria at genus level (red circles) in the ileum. Silver lines, negative correlation (*p* < 0.05); Golden lines, positive correlation (*p* < 0.05).

Since the above GSEA algorithm of transcriptomes results releveled potential core genes (enriched in positive regulation of MAPK cascade and regulation of I-kappaB kinase/NF-kappaB signaling) associated with immune responses only in the ileum, rather than the rumen, the spearman’s correlation coefficient was used to investigate the relationship between serum cytokines and dominant bacterial genera (top 20) in the ileum. Correlation analysis showed that *Clostridium_XI* was negatively correlated with VEGF, TGF-β, TNF-α, and HGF (*p* < 0.05). Likewise, Escherichia showed a negative correlation with IL-2, VEGF, TGF-β, TNF-α, and HGF (*p* < 0.05) (Figure 4). Moreover, the correlation between the core genes enriched by positive regulation of MAPK cascade and regulation of I-kappaB kinase/NF-kappaB signaling in GSEA and the dominant bacterial genera in ileum were analyzed. The results showed that the core genes *BMP4* and *CD4* were negatively correlated with *Clostridium_XI* (*p* < 0.05) (Figure 5).

## 4. Discussion

The rapidly growing livestock sector and the increasing demand for livestock feeds have led to a substantial upsurge in feedstuff prices, especially protein feed resources. Finding alternative protein feed resources that could replace commercial protein supplements without profoundly affecting growth performance and health is required. Herein, we performed a comprehensive evaluation of silybum marianum meal as a potential replacement for soybean meal in growing lambs using a multi-omics approach. In this study, neither growth performance nor feed intakes were affected by the replacement of soybean meal with silybum marianum meal, suggesting that silybum marianum meal could be used in the diets of growing lambs. The inclusion of a higher percentage of silybum marianum meal in the diets of broilers has been reported to limit the body weight gain of broilers due to the high content of fiber and nitrite in silybum marianum meal (Suchý et al., 2008). However, ruminants can effectively utilize the high fiber and nitrite content of silybum marianum meal (Niyas et al., 2019) without adversely affecting their growth. In this regard, it has been reported that adding 20% *silymarin* flour to the diet of buffaloes improved the digestibility of NDF to some extent (Nikzad et al., 2017).

The silybum marianum meal usually contains 2–4% active substances on a dry matter basis (Stastnik et al., 2020). It was expected that the *silymarin* complex or flavonoid in the silybum marianum meal could induce the immune responses of animals. Cytokines are proteins or small polypeptides with immunoregulatory and effector functions, including lymphokines, monokines, various growth factors and others (Matthews, 2002). The stimulating and releasing of pro-inflammatory cytokines are essential steps for the activation of innate host defenses and subsequently regulating adaptive immune responses (Netea et al., 2010). In this study, the elevated pro-inflammatory factors IL-1β and TNF-α in SIL group were mainly resulted from its low crude protein content, low palatability (Stastnik et al., 2020) and high nitrite (Arviv et al., 2016). However, if there is enough energy feed in the rumen (such as corn), nitrite can be easily converted into ammonia and eventually used for microbial protein synthesis for ruminants (Niyas et al., 2019). TGF- β has been reported to suppress the immune system by inhibiting the immune function of inflammatory cells and promoting the differentiation and function of Treg cells (Pourgholaminejad et al., 2016). In our study, the increased level of TGF- βin the SIL group may inhibit excessive increase of proinflammatory factors and maintain the balance of the immune function. Moreover, it has been reported that silymarin has anti-inflammatory properties (Hadolin et al., 2001) and enhances cell growth factors (Vahabzadeh et al., 2018). The unregulated HGF in the SIL group in our study was expected, since TGF-β can exhibit immunosuppressive effects in synergy with HGF (Okunishi et al., 2005). We found that both the pro-inflammatory and anti-inflammatory cytokines were upregulated in lambs fed silybum marianum meal, which may indicate an immune system balance and result in minimal effects in growth performance and biochemical parameters of growing lambs.

Since the observed number of differential genes in the rumen and ileum was small (Supplementary file 4), a further GSEA was conducted to detect subtle gene expression changes. Fructose and mannose metabolisms are vital in cellular productivity, complex cellular processes (e.g., glycosylation) and cellular health (Lieu et al., 2021). In the present study, the GSEA analysis showed that feeding silybum marianum meal to growing lambs upregulated some genes in the fructose and mannose metabolism pathway in the rumen. This implies that the use of silybum marianum meal as a component in the growing lamb is beneficial for rumen carbohydrate metabolism. In the ileum, it was found that three biological process-related genes, including digestive tract development, positive regulation of MAPK cascade, and regulation of I-kappaB kinase/NF-kappaB signaling, were upregulated in the SIL group. MAPK cascade activation is central to various signaling pathways that regulate various important cellular physiological processes such as cell growth, differentiation, adaptation to environmental stress, and inflammatory responses (Sun et al., 2015; Kirk et al., 2020). NF-kappaB has a normal physiological function in mediating the immune response. The inflammatory response of the organism, after infection, requires the initiation of NF-kappaB signaling pathway to transcribe some cytokines to mediate the immune response to clear the invading pathogenic bacteria (Sun, 2017; Wang and Shen, 2022). Our results indicated that the ileum was the more important section for immune functions in response to feeding silybum marianum meal as compared with the rumen.

Ruminal microorganisms play a crucial role in feed degradation and production of volatile fatty acids, lipids, amino acids and hydrogen, which are essential for ruminants to maintain growth and productive performance (Kim et al., 2009; Lopes et al., 2015; Salami et al., 2021). Changes in diet composition or type can alter the microbial structure and composition in the rumen (Ramos et al., 2021). Our study found a higher microbial diversity in the rumen of lambs from the SIL group, as evidenced by the alpha and beta diversity indexes. These changes might be associated with the high fiber content in the silybum marianum meal (Koh et al., 2016). In ruminants, *Bacteroidetes* are mainly responsible for the degradation of cellulose, hemicellulose and pectin, and *Firmicutes* are mainly responsible for the decomposition of exogenous peptides and amino acids (Cholewińska et al., 2021). In our study, the relative abundance of *Bacteroidetes* and *Firmicutes* phyla in the rumen of lambs from the SIL group was higher which was in line with reports for the gut of healthy lambs (Gebeyew et al., 2021). However, the relative abundance of *Sharpea* belongs to *Firmicutes* was decreased in the SIL group, which was reported to be enriched in the low-methane producing sheep (Kamke et al., 2016). Studies have found a positive association of *Proteobacteria* with rumen biohydrogenation in ruminants (Salami et al., 2021). The lower relative abundance of *Proteobacteria* in the SIL group suggested that replacing soybean meal with silymarin marianum meal may alter the ruminal biohydrogenation. The phylum *Synergistetes* is generally found in the gut as a minor component, where it co-metabolizes secondary phytocompounds (Jumas-Bilak et al., 2009). In addition, Kang et al. (2020) found that *Pyramidobacter* from *Synergistetes* phylum can degrade natural toxic substances in plants. Interestingly, the relative abundance of *Synergistetes* phylum and *Pyramidobacter* genera in the SIL group was higher in the present study, which may be associated with the presence of some secondary compound in the silybum marianum meal. *Saccharofermentans*, as sugar fermenters, are able to ferment glucose, D-fructose, esculin, sucrose, starch, galactitol, mannitol, myo inositol and adonitol (Chen et al., 2010). In our study, the LEfSe analysis showed that *Bacteroidetes*, *Firmicutes*, *Synergistetes*, *Verrucomicrobia*, *Pyramidobacter*, and *Saccharofermentans* were enriched in the rumen of the SIL group compared with the CON group, whereas the *Proteobacteria* and *Sharpea* were depleted. These differential microorganisms further suggested that silybum marianum meal can maintain the nutrient and growth needs for growing lambs.

The ileum is an important site for the digestion and absorption of nutrients such as lipids, bile salts, and vitamins (Wang et al., 2020). In addition, the ileum also gathers a relatively large number of immune-related cells and tissues, such as Paneth cells (producing antimicrobial peptides, such as lysozyme, defensins and other antibacterial substances) and Peyer’s patches (gut-associated lymphoid tissue) (Mowat and Agace, 2014), making it as an important part of the body’s resistance to pathogens. The innate and acquired immune function of the ileum have been reported to be significantly affected by gut microorganisms (Martinez-Guryn et al., 2019). It has been reported that the addition of 3% silybum marianum meal was able to reduce the count of *E. coli* in the ileum of laying hens (Hashemi Jabali et al., 2018). In our study, the abundance of *Clostridium_XI* was lower in the ileum of lambs from the SIL group, which was probably due to the higher fiber content in the silybum marianum meal. In this regard, Zheng et al. (2018) have indicated that feeding fermentable fiber reduced the presence of *Clostridium_XI* in the intestine of mice. In addition, our enriched abundances of *Bifidobacterium* and *Ruminococcus* genera in SIL group implied a healthier ileum environment because of the positive effect of *Bifidobacterium* and *Ruminococcus* on intestine health (Belkaid and Hand, 2014; Bi et al., 2019; Ding et al., 2020).

Integrated transcriptome and microbial community analysis in the ileum was further conduced to explore the potential mechanisms for increased blood cytokines in SIL group. Initially, it was speculated that silybum marianum meal resulted in stress in lambs, as evidenced by the enrichment of genes related to MAPK cascade and NF-kappaB signaling identified using GSEA and the production of cytokines. The enriched core genes related to MAPK cascade and NF-kappaB signaling in the ileum, including *BMP4* (Hogg et al., 2010), *P2RX7*, *CD4* (Blanc et al., 2018), *HSPB1* (Lee et al., 2012), were expected to be involved in cytokine production. Moreover, it was observed that *Clostridium_XI* was as the dominant genus in the ileum in the present study. It has been reported that *Clostridium_XI* produces toxins that could induce intestinal damage (Wan et al., 2022). In the present study, the abundance of *Clostridium_XI* was depleted in the SIL group compared to the CON group. It would be explainable that the increases in cytokines levels may be associated with the colonization of *Clostridium_XI*. It has been reported that toxins produced by *Clostridium_XI* triggered circulating immunity to increase the expression of some interleukins, such as IL-1β, prompting immune cells to secrete antimicrobial peptides in an attempt to clear *Clostridium_XI* from the gut (Hernández Del Pino et al., 2021; Nibbering et al., 2021). This statement coincided with our negative correlation between *Clostridium_XI* and blood cytokines. It was speculated that feeding silybum marianum meal to lambs may drive MAPK cascade and NF-kappaB signaling-related gene enrichment in the ileum, which may in turn produce cytokines that mediate a decrease in the abundance of the harmful bacterium, such as *Clostridium_XI.*

## 5. Conclusion

Replacement of soybean meal with silybum marianum meal in the diet of lambs resulted in increased cytokines production without affecting growth performance, feed intakes or blood biochemical parameters in growing lambs. Feeding silybum marianum meal as a replacement for soybean meal differently altered the transcriptome profiles and microbial community in the rumen and ileum. Further integrated transcriptome and microbial community analysis in the ileum revealed that silybum marianum meal promoted the enrichment of immune-related genes (such as *BMP4* and *CD4*), which were supposed to trigger the production of cytokines and suppress the abundance of harmful bacteria, such as *Clostridium_XI.*

## Data availability statement

The data presented in the study are deposited in the NCBI Sequence Read Archive (SRA) database repository, accession number PRJNA919487 (ileal tissue transcriptome), accession number PRJNA918960 (rumen tissue transcriptome), accession number PRJNA917096 (ileal digesta 16s rDNA), accession number PRJNA917525 (rumen digesta 16s rDNA).

## Ethics statement

The experimental protocols were reviewed and approved by the Animal Care and Use Committee of the Institute of Subtropical Agriculture, Chinese Academy of Sciences (ISA-202020) and all applicable international, national, and/or institutional guidelines for the care and use of animals were followed.

## Funding

This study was funded by Chinese Academy of Sciences (Strategic Priority Research Program Grant Nos. XDA26040304 and XDA26050102), the National Natural Science Foundation of China (32072760), and the Natural Science Foundation of Hunan Province of China (2022JJ10054), and Innovation Province Project (2019RS3021).

## Author contributions

TZ: conceptualization, methodology, data acquisition, data analysis, and writing–original draft. YR: and TZ: animal experiments and data acquisition. CY, KG, and MG: review and editing. ZH: conceptualization, methodology, project administration, supervision, and review and editing. ZT: conceptualization, methodology, and project administration. All authors contributed to the article and approved the submitted version.

## Conflict of interest

The authors declare that the research was conducted in the absence of any commercial or financial relationships that could be construed as a potential conflict of interest.

## Publisher’s note

All claims expressed in this article are solely those of the authors and do not necessarily represent those of their affiliated organizations, or those of the publisher, the editors and the reviewers. Any product that may be evaluated in this article, or claim that may be made by its manufacturer, is not guaranteed or endorsed by the publisher.
