# Supplementary material for: An integrated transcriptome and microbial community analysis reveals potential mechanisms for increased immune responses when replacing silybum marianum meal with soybean meal in growing lambs
Source: Front Microbiol. 2023 Mar 2;14:1093129. doi: 10.3389/fmicb.2023.1093129 (PMC10018209; doi:10.3389/fmicb.2023.1093129)
Supplement: Supplementary file 1 [file Table_1.DOCX]

**Supplementary material**

**Supplementary Table 1** The relative abundance of bacteria in the rumen digesta samples at phylum level.

| Phylum | Treatment | | SEM | *P-*value |
| --- | --- | --- | --- | --- |
|  | CON  (n=7) | SIL  (n=7) |  |  |
| *Proteobacteria* | 39.768^a^ | 14.098^b^ | 4.69 | 0.009 |
| *Bacteroidetes* | 39.711^b^ | 52.272^a^ | 2.95 | 0.024 |
| *Firmicutes* | 16.328^b^ | 25.606^a^ | 2.41 | 0.018 |
| *Spirochaetes* | 3.122 | 4.271 | 0.70 | 0.385 |
| *Synergistetes* | 0.344^b^ | 1.202^a^ | 0.21 | 0.013 |
| *Elusimicrobia* | 0.060 | 0.301 | 0.12 | 0.102 |
| *Candidatus_Saccharibacteria* | 0.046 | 0.131 | 0.03 | 0.118 |
| *Fibrobacteres* | 0.045 | 0.079 | 0.03 | 0.414 |
| *Actinobacteria* | 0.031 | 0.101 | 0.02 | 0.272 |
| *Verrucomicrobia* | 0.023^b^ | 1.007^a^ | 0.24 | 0.009 |
| *Tenericutes* | 0.010 | 0.060 | 0.02 | 0.056 |
| *Lentisphaerae* | 0.006 | 0.009 | 0.002 | 0.953 |
| *SR1* | 0.004 | 0.001 | 0.002 | 0.620 |
| *Fusobacteria* | 0.001 | 0.003 | 0.001 | 0.028 |

CON= supplemented with 10% soybean meal; SIL= supplemented with 10% silybum marianum meal.

Values are expressed as means ± SEM, n = 7. *P* < 0.05 was regarded as statistically significant, and 0.05 < *P* < 0.10 was regarded as a statistical tendency.

^a,b^ Means with different superscripts differ (*P*< 0.05).

**Supplementary Table 2** The relative abundance of bacteria in the rumen digesta samples at genus level (average relative abundance > 0.1% in at least one group)

| Genus | Treatment | | SEM | *P*-value |
| --- | --- | --- | --- | --- |
|  | CON | SIL |  |  |
| *Prevotella* | 36.061 | 34.338 | 3.438 | 0.862 |
| *Treponema* | 3.106 | 3.920 | 0.732 | 0.603 |
| *Ruminococcus* | 2.480 | 2.151 | 0.591 | 0.603 |
| *Succiniclasticum* | 2.390 | 3.033 | 0.325 | 0.148 |
| *Bacteroides* | 1.401 | 0.613 | 0.299 | 0.452 |
| *Butyrivibrio* | 0.826 | 0.443 | 0.172 | 0.325 |
| *Lachnospiracea_incertae_sedis* | 0.746 | 0.611 | 0.128 | 0.452 |
| *Succinivibrio* | 0.742 | 6.008 | 2.703 | 0.954 |
| *Sharpea* | 0.444^a^ | 0.283^b^ | 0.199 | 0.042 |
| *Mitsuokella* | 0.388 | 0.307 | 0.191 | 0.183 |
| *Pyramidobacter* | 0.343^b^ | 0.962^a^ | 0.146 | 0.018 |
| *Selenomonas* | 0.324 | 0.592 | 0.041 | 0.954 |
| *Kandleria* | 0.136 | 0.032 | 0.036 | 0.052 |
| *Saccharofermentans* | 0.135^b^ | 0.859^a^ | 0.034 | 0.043 |
| *Desulfovibrio* | 0.116 | 0.350 | 0.064 | 0.118 |
| *Holdemania* | 0.111 | 0.108 | 0.034 | 0.603 |
| *Eubacterium* | 0.073 | 0.209 | 0.035 | 0.024 |
| *Megasphaera* | 0.070 | 0.135 | 0.040 | 0.772 |
| *Elusimicrobium* | 0.060 | 0.206 | 0.075 | 0.161 |
| *Saccharibacteria* | 0.046 | 0.131 | 0.002 | 0.118 |
| *Mogibacterium* | 0.032 | 0.116 | 0.027 | 0.118 |
| *Moryella* | 0.031 | 0.111 | 0.036 | 1.000 |
| *Clostridium_XlVa* | 0.027 | 0.190 | 0.009 | 0.073 |
| *Anaerovibrio* | 0.022 | 0.362 | 0.095 | 0.018 |
| *Sphaerochaeta* | 0.015 | 0.318 | 0.169 | 0.073 |
| *Oscillibacter* | 0.015 | 0.169 | 0.042 | 0.005 |
| *Pseudobutyrivibrio* | 0.011 | 0.204 | 0.046 | 0.003 |
| *Olivibacter* | 0.006 | 8.634 | 3.462 | 0.170 |
| *Barnesiella* | 0.005 | 0.485 | 0.146 | 0.006 |
| *Ruminobacter* | 0.001 | 0.244 | 0.084 | 0.077 |
| *Fretibacterium* | 0.001 | 0.234 | 0.115 | 0.009 |

CON= supplemented with 10% soybean meal; SIL= supplemented with 10% silybum marianum meal.

Values are expressed as means ± SEM, n = 7. *P* < 0.05 was regarded as statistically significant, and 0.05 < *P* < 0.10 was regarded as a statistical tendency.

^a,b^ Means with different superscripts differ (*P*< 0.05).

**Supplementary Table 3** The relative abundance of bacteria in the ileum digesta samples at phylum level.

| Phylum | Treatment | | SEM | *P*-value |
| --- | --- | --- | --- | --- |
|  | CON  (n=7) | SIL  (n=7) |  |  |
| *Firmicutes* | 90.098 | 67.867 | 7.095 | 0.121 |
| *Proteobacteria* | 3.424 | 3.322 | 0.822 | 0.954 |
| *Tenericutes* | 1.995 | 7.837 | 3.255 | 0.391 |
| *Bacteroidetes* | 1.807 | 13.867 | 4.539 | 0.195 |
| *Unclassified* | 0.885 | 1.312 | 0.297 | 0.494 |
| *Candidatus_Saccharibacteria* | 0.513 | 0.729 | 0.323 | 0.753 |
| *Actinobacteria* | 0.361 | 1.078 | 0.417 | 0.411 |
| *Verrucomicrobia* | 0.302 | 1.446 | 0.511 | 0.280 |
| *Chlamydiae* | 0.175 | 0.362 | 0.169 | 0.599 |
| *Elusimicrobia* | 0.147 | 1.217 | 0.583 | 0.380 |
| *Spirochaetes* | 0.136 | 0.847 | 0.259 | 0.179 |
| *Lentisphaerae* | 0.079 | 0.031 | 0.017 | 0.169 |
| *Synergistetes* | 0.056 | 0.075 | 0.035 | 0.799 |
| *Fibrobacteres* | 0.023 | 0.002 | 0.009 | 0.265 |
| *Fusobacteria* | 0.001 | 0.003 | 0.001 | 0.431 |
| *Deferribacteres* | 0.001 | 0.007 | 0.002 | 0.183 |

CON= supplemented with 10% soybean meal; SIL= supplemented with 10% silybum marianum meal.

Values are expressed as means ± SEM, n = 7. *P* < 0.05 was regarded as statistically significant, and 0.05 < *P* < 0.10 was regarded as a statistical tendency.

^a,b^ Means with different superscripts differ (*P*< 0.05).

**Supplementary Table 4** The relative abundance of bacteria in the rumen digesta samples at genus level (average relative abundance > 0.1% in at least one group)

| Genus | Treatment | | SEM | *P*-value |
| --- | --- | --- | --- | --- |
|  | CON | SIL |  |  |
| *Romboutsia* | 33.347 | 22.101 | 3.919 | 0.159 |
| *Unclassified* | 26.756 | 33.516 | 3.588 | 0.367 |
| *Clostridium_XI* | 20.609^a^ | 7.627^b^ | 2.658 | 0.008 |
| *Clostridium_sensu_stricto* | 4.872 | 9.738 | 3.190 | 0.468 |
| *Kandleria* | 1.798 | 0.022 | 0.819 | 0.296 |
| *Yersinia* | 1.513 | 1.078 | 0.629 | 0.744 |
| *Mycoplasma* | 1.421 | 2.583 | 1.288 | 0.670 |
| *Succiniclasticum* | 1.264 | 0.134 | 0.622 | 0.385 |
| *Turicibacter* | 1.009 | 0.364 | 0.265 | 0.238 |
| *Escherichia* | 0.852 | 0.124 | 0.312 | 0.258 |
| *Mogibacterium* | 0.700 | 0.426 | 0.250 | 0.605 |
| *Holdemania* | 0.588 | 0.453 | 0.222 | 0.774 |
| *Saccharibacteria* | 0.513 | 0.729 | 0.323 | 0.753 |
| *Ureaplasma* | 0.511 | 5.161 | 2.045 | 0.272 |
| *Bacteroides* | 0.320 | 4.485 | 1.496 | 0.173 |
| *Prevotella* | 0.316 | 1.257 | 0.580 | 0.440 |
| *Akkermansia* | 0.282 | 0.370 | 0.173 | 0.809 |
| *Olsenella* | 0.264 | 0.065 | 0.056 | 0.072 |
| *Lachnospiracea_incertae_sedis* | 0.259 | 0.125 | 0.068 | 0.347 |
| *Succinivibrio* | 0.238 | 0.092 | 0.114 | 0.543 |
| *Campylobacter* | 0.209 | 0.308 | 0.086 | 0.588 |
| *Cellulosilyticum* | 0.194 | 0.088 | 0.049 | 0.297 |
| *Butyrivibrio* | 0.177 | 0.141 | 0.066 | 0.795 |
| *Chlamydophila* | 0.175 | 0.362 | 0.169 | 0.599 |
| *Eubacterium* | 0.166 | 0.586 | 0.148 | 0.164 |
| *Sharpea* | 0.149 | 0.069 | 0.044 | 0.388 |
| *Elusimicrobium* | 0.147 | 1.217 | 0.583 | 0.380 |
| *Treponema* | 0.131 | 0.732 | 0.244 | 0.231 |
| *Alistipes* | 0.124 | 0.478 | 0.156 | 0.272 |
| *Bulleidia* | 0.108 | 0.019 | 0.024 | 0.057 |
| *Clostridium_XlVa* | 0.085 | 0.780 | 0.257 | 0.187 |
| *Desulfovibrio* | 0.065 | 0.236 | 0.061 | 0.169 |
| *Phascolarctobacterium* | 0.042 | 0.332 | 0.112 | 0.204 |
| *Oscillibacter* | 0.037 | 0.272 | 0.088 | 0.194 |
| *Ruminococcus* | 0.035^b^ | 0.285^a^ | 0.058 | 0.023 |
| *Intestinimonas* | 0.029 | 0.149 | 0.035 | 0.083 |
| *Paraprevotella* | 0.029 | 0.877 | 0.296 | 0.160 |
| *Anaerotruncus* | 0.025 | 0.167 | 0.054 | 0.195 |
| *Saccharofermentans* | 0.022 | 0.157 | 0.044 | 0.130 |
| *Clostridium_XlVb* | 0.015 | 0.185 | 0.061 | 0.171 |
| *Bifidobacterium* | 0.003^b^ | 0.607^a^ | 0.276 | 0.017 |
| *Lawsonia* | 0.002 | 0.190 | 0.095 | 0.342 |
| *Faecalicoccus* | 0.000 | 0.126 | 0.043 | 0.157 |

CON= supplemented with 10% soybean meal; SIL= supplemented with 10% silybum marianum meal.

Values are expressed as means ± SEM, n = 7. *P* < 0.05 was regarded as statistically significant, and 0.05 < *P* < 0.10 was regarded as a statistical tendency.

^a,b^ Means with different superscripts differ (*P*< 0.05).
